# Editorial: Relevance of Translational Regulation on Plant Growth and Environmental Responses

**DOI:** 10.3389/fpls.2017.02170

**Published:** 2017-12-19

**Authors:** Alejandro Ferrando, M. Mar Castellano, Purificación Lisón, Dario Leister, Anna N. Stepanova, Johannes Hanson

**Affiliations:** ^1^Instituto de Biología Molecular y Celular de Plantas, Consejo Superior de Investigaciones Científicas-Universitat Politècnica de València, Valencia, Spain; ^2^Centro de Biotecnología y Genómica de Plantas, Universidad Politécnica de Madrid - Instituto Nacional de Investigación y Tecnología Agraria y Alimentaria, Madrid, Spain; ^3^Plant Molecular Biology, Department Biology I, Ludwig-Maximilians-Universität München, Planegg-Martinsried, Germany; ^4^Genetics Graduate Program, Department of Plant and Microbial Biology, North Carolina State University, Raleigh, NC, United States; ^5^Department of Plant Physiology, Umeå Plant Science Centre, Umeå University, Umeå, Sweden

**Keywords:** mRNA translation, translation factors, post-transcriptional regulation, translatome, organellar gene expression

One of the great challenges in the near future will be the sustainable production of sufficient amounts of safe food worldwide. A combination of adverse demographic factors and climatological perturbations is expected to impact on food systems globally (Vermeulen et al., 2012). To ensure food security in the coming years, multidisciplinary approaches are needed, and useful leads are likely to emerge from advances in plant biotechnology. However, improving plants' performance under restrictive growth conditions will require a deep understanding of the molecular processes that underlie their extraordinary physiological plasticity.

Much research in plant biology in recent decades has focused on phenomenological descriptions of changes in gene expression at the mRNA level. However, in eukaryotes, shifts in transcript levels do not always correlate with equivalent changes in protein levels, since a variety of post-transcriptional events may uncouple transcription from translation (Vogel and Marcotte, 2012). This is particularly relevant in plants, given the intrinsically complex properties of their translational apparatus, the presence of additional genetic systems in mitochondria and chloroplasts, and the occurrence of environment-dependent variation of cytosolic ribosome composition (Hummel et al., 2012). Recent advances in *next-generation-sequencing*, combined with breakthrough technologies like *ribosome footprint profiling*, may help to close the gap between transcriptional and translational studies and further elucidate plant responses to environmental factors.

In this Research Topic, we present reviews and original research articles that extend our knowledge of post-transcriptional and translational mechanisms that regulate the production of plant proteins which ultimately execute the cellular functions required for adaptation to environmental challenges.

Many of the contributions highlight the importance of translational regulation. Among the panoply of translational regulators, the TOR kinase emerges as a key regulatory element. Dobrenel et al. have used transgenic plants deficient in TOR activity to reveal its role in regulating levels of chloroplast ribosomal proteins, possibly by recognizing a sequence motif present in their mRNAs. This work also demonstrated that the TOR pathway is involved in phosphorylation of the ribosomal protein S6. Two reviews summarize our growing knowledge of the roles of the TOR signaling pathway in plants. Schepetilnikov and Ryabova review recent findings that link auxin signaling to TOR kinase activity via the small GTPase ROP2, and explain how this pathway contributes to translational regulation of mRNAs that harbor upstream ORFs within their 5′-leader region that inhibit translation of the main ORF. Sesma et al. describe recent advances in the regulation of TOR signaling, eIF4E activity and eIF2α phosphorylation in plants. These authors highlight the paucity of our knowledge of the regulation of these important players in plants and the need for further studies to clarify the relevance of controls on translational initiation.

Another key regulator of translational initiation is the large protein complex eIF3. Wang et al. have investigated the physiological effects of reduced expression of the eIF3e subunit in transgenic rice plants. These transgenics grew more slowly and remained smaller in size than controls, showed impaired pollen maturation and were more sensitive to osmotic stress. The results indicate that eIF3e plays surprisingly specific roles in plant growth and development.

One of the key post-transcriptional steps in gene expression is the regulation of mRNA levels by microRNAs (miRNAs). Several studies have demonstrated that abiotic stress conditions induce aberrant expression of miRNAs that reduce steady-state levels of their target mRNAs. To shed more light on this subtopic Li et al. have used high-throughput sequencing and computational approaches to identify a large number of stress-related miRNAs involved in melatonin-mediated cold tolerance in watermelon. In a similar way Aravind et al. have studied inbred lines of subtropical maize to identify miRNAs involved in drought stress.

Two papers included in the Topic deal with aspects of organellar gene expression. Leister et al. review the link between the expression of chloroplast genes and whole-cell acclimation to environmental changes. Although only a small fraction of the genes present in the original cyanobacterial endosymbiont remains in the modern organelle, perturbation of their expression plays a major role in triggering acclimation and tolerance responses via signaling from the chloroplast to the nucleus. Zoschke et al. have investigated the effect of a maize chlorophyll-deficient mutant, *chl1H/gun5*, on the translation of plastidic transcripts coding for chlorophyll-binding apoproteins (CBPs). By comparing the positions and numbers of ribosomes on the plastidic transcripts of wild-type and mutant plants, the authors concluded that chlorophyll availability modulates the stability rather than the synthesis of CBPs in plastids. Furthermore, the *chl1H* mutation had no effect on the partitioning of CBP footprints, suggesting that co-translational targeting of the nascent peptides into the thylakoid membrane is independent of chlorophyll binding by the CBPs.

Finally, the Topic includes a review of translational regulation during development and under stressful conditions, and an overview of the translation of viral RNAs. Sablok et al. summarize recent global analyses of mRNA populations associated with ribosomes (now referred to as the “translatome”), highlighting the importance of alternative splicing and the application of these technologies to polyploid plant species. Miras et al. focus on translation in plants infected with RNA viruses. Plant viruses have evolved subtle mechanisms, such as mRNA *cis*-translational enhancers, to recruit the host's translational machinery and initiate translation by non-canonical mechanisms. The authors highlight the diversity of these translational elements and focus on current knowledge of their structure and interactions with the host's translational initiation apparatus.

We believe that this compilation of original research articles and reviews will bring the reader up to date on the current state of the art in the field of post-transcriptional and translational regulation in plants. We are also convinced that advances in this area will be of the utmost importance for the development of biotechnological tools for yield enhancement.

## Author contributions

All authors listed have made substantial, direct and intellectual contribution to the work, and approved it for publication.

### Conflict of interest statement

The authors declare that the research was conducted in the absence of any commercial or financial relationships that could be construed as a potential conflict of interest.
